# A Novel Magnetic Molecular Imprinted Polymer for Selective Extraction of Zearalenone from Cereal Flours before Liquid Chromatography-Tandem Mass Spectrometry Determination

**DOI:** 10.3390/toxins11090493

**Published:** 2019-08-27

**Authors:** Chiara Cavaliere, Michela Antonelli, Andrea Cerrato, Giorgia La Barbera, Aldo Laganà, Michele Laus, Susy Piovesana, Anna Laura Capriotti

**Affiliations:** 1 Department of Chemistry, Università di Roma “La Sapienza”, 00185 Rome, Italy; 2 CNR, NANOTEC – Campus Ecotekne, Università del Salento, 73100 Lecce, Italy; 3 Department of Science and Technological Innovation, Università del Piemonte Orientale “A. Avogadro”, 15121 Alessandria, Italy

**Keywords:** dispersive solid-phase extraction, dummy template, liquid chromatography-tandem mass spectrometry, magnetic molecularly imprinted polymers, sample preparation, zearalenone

## Abstract

Zearalenone (ZEN) is a nonsteroidal estrogenic mycotoxin produced by various *Fusarium* species and commonly occurring in corn and other cereals. Even though its acute toxicity is low, still the estrogenic activity of ZEN and metabolites is a matter of concern. In this work, a new magnetic molecularly imprinted polymer (mMIP) for the selective extraction of ZEN from cereal flours is presented. The mMIP was synthesized previously using quercetin as dummy template, and here we wanted to test its applicability to complex food samples. Analyte determination was carried out by high-performance liquid chromatography coupled to tandem mass spectrometry. The selectivity of the mMIP and the main validation method parameters were assessed. In particular, even in samples as complex as cereals, matrix effect was negligible. Although the mMIP showed cross-selectivity towards both ZEN-related and quercetin-related compounds, nonetheless ZEN recovery was > 95% for the two lower spiking levels, and the quantification limit was 0.14 ng g^−1^, i.e., ca. 500 times lower than the maximum limit fixed for most cereals by European law. Therefore, the material, also in comparison with a commercial sorbent, appears suitable for the application in food analysis, also to isolate ZEN at trace levels.

## 1. Introduction

**Figure 1:**
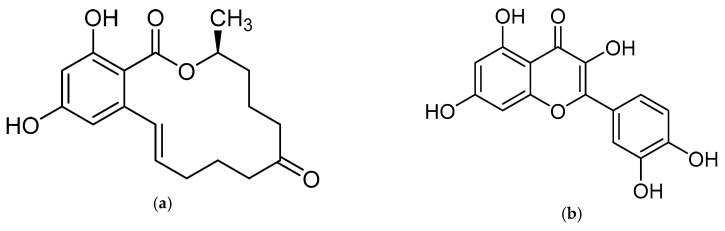
(**a**) Zearalenone; (**b**) Quercetin used as a dummy template in the synthesis of a molecularly imprinted polymer for zearalenone.

The mycotoxin zearalenone (ZEN) is a resorcylic acid lactone (see Figure 1a) produced by several species of *Fusarium* genus, such as *Fusarium roseum, Fusarium tricinctum, Fusarium sporotrichioides*, *Fusarium oxysporum*, and *Fusarium moniliforme* [1]. In temperate and warm countries, these *Fusarium* species may colonize various cereal grains, such as maize, sorghum, wheat, barley, and oats [2], mainly in the field but also post-harvesting under poor storage conditions, e.g., high humidity.

Despite the low acute toxicity of ZEN, with oral 50% lethal dose values ranging between 2 and 20 g kg^−1^ body weight (bw) depending on the tested species [3], still, the estrogenic activity of ZEN and its metabolites and derivatives is well recognized at much lower levels [2,4]. Indeed, for a correct risk assessment, it should be important to take into account not only the parent compound but also its modified forms, which comprise all metabolites formed in the fungus, infested plant, and mammalian organism [5,6]. Thus, ZEN, α- and β-zearalenol (α/β-ZEL), α- and β-zearalanol (α/β-ZAL), and zearalanone (ZAN) are classified as mycoestrogens [7]; they act by binding estrogen receptors in a rank order which, for animals, is α-ZEL > α-ZAL > ZEN ≈ ZAN ≈ β-ZAL > β-ZEL [4,6]. Other reported toxic effects of ZEN include hepatotoxicity, haematotoxicity, immunotoxicity, and genotoxicity.

In the European Union, maximum levels (MLs) for certain contaminants in foodstuffs, including ZEN, have been fixed [8]. For cereals, cereal flour, bran, and germ intended for direct human consumption, the ML is 75 µg kg^−1^, whereas a slightly higher value (100 µg kg^−1^) is set for maize intended for direct human consumption and unprocessed cereals other than maize. A much higher value is fixed for unprocessed maize (350 µg kg^−1^), whereas, for young children and baby food, the ML is significantly lower (20 µg kg^−1^). EFSA Panel for Contaminants in the Food Chain established a tolerable daily intake for ZEN (expressed as ZEN equivalents for ZEN and its modified forms) of 0.25 µg kg^−1^ bw based on estrogenicity in pigs [6]. In a data collection about ZEN contamination in food in some European countries, ZEN was reported above the limit of quantification (LOQ) in 15% of the samples, with the highest concentrations observed in wheat bran, corn, and products thereof (e.g., cornflour, cornflakes) [9]. The total chronic dietary exposures to ZEN was estimated, and, in all age classes, the largest contribution was due to grains and grain-based foods, in particular grains and grain milling products, bread, and fine bakery wares.

For mycoestrogen determination, after high-performance liquid chromatography (HPLC) separation, either fluorescence and mass spectrometry (MS) detection are used [9]. Nevertheless, MS is preferred, in particular in multitoxin methods [10,11], because of its higher selectivity, thus requiring less extensive sample clean-up (up to the “dilute and shoot” approach).

Generally, ZEN and its metabolites are extracted from cereals by using solvent mixtures, such as acetonitrile/water [12,13], acetonitrile/water/acetic acid [14,15,16], methanol/water [17], following the “dilute and shoot” approach [15] or adding a clean-up step based on different solid-phase extraction (SPE) sorbents [12,13,16,17], including immunoaffinity columns, to improve selectivity and reduce matrix effect (ME). Indeed, selectivity is often the goal when dealing with contaminants at such low levels in complex food matrices. For this reason, molecularly imprinted polymers (MIPs) have been proposed for ZEN selective binding [18,19,20,21] since these synthetic materials can specifically rebind a target molecule even in the presence of related compounds.

There are various approaches to prepare MIPs; however, the main steps are: (i) formation of a pre-polymerization complex between the template molecule and the monomer; (ii) polymerization with a cross-linker; (iii) template removal [22]. Then, MIP binding efficiency is generally assessed by comparison with the corresponding not imprinted polymer (NIP), i.e., a polymer synthesized in the same way but in the absence of the template. One of the main drawbacks of MIPs could be the template bleeding from the polymer, but it can be overcome by using a dummy template [23], which is ideally an isotope-labeled compound of the target molecule, a fragment or a structurally related analog. Thus, MIPs for ZEN selective binding have been prepared using quercetin (QUE) [19] and cyclododecyl 2,4-dihydroxybenzoate [20,21] as dummy templates.

Recently, there is an increasing interest towards techniques derived from SPE, such as solid-phase microextraction, dispersive SPE, and magnetic SPE [22]; magnetic MIPs (mMIPs) have been prepared too [24]. Starting from all these considerations, we wanted to test the capability of a magnetic material constituted by a MIP imprinted with QUE (Figure 1b) for the selective extraction of ZEN from cereal flours. This polymer was prepared and characterized in previous work [25], whereas, here, we wanted to demonstrate its applicability in food matrices. The performance of this new magnetic material was assessed and, finally, the mMIP was compared with a widespread commercial SPE sorbent.

## 2. Results and Discussion

### 2.1. Preparation of mMIP

The rationale was to use a dummy template for synthesizing a polymer selective towards ZEN. The employment of a dummy template is a well-consolidated approach to overcome inaccuracy in target analyte quantitation and other inconvenience due to template bleeding from the polymer [23]; however, the choice of a dummy template different from a labeled compound is often critical since this can limit the MIP selectivity. QUE was chosen as a dummy template following the work by Weiss et al. [19], who used the copolymer 4-vinylpyridine (4-VP)-ethyleneglycol dimethacrylate (EDMA) as HPLC stationary phase for ZEN separation. They argue that in the preparation of the polymer imprinted with ZEN, the incorporation of the template could likely occur due to the reactivity of the double bond in the ring system of ZEN, which in turn hinders the removal of the template and blocks the binding sites. Furthermore, the ZEN macrocyclic flexible ring structure inhibits an exactly fitting binding site during the MIP synthesis. Ideally, ZAN and ZAL could be used as templates, but cost and safety should be taken into account. The structural similarity between ZEN and QUE mainly lies in the two hydroxyl groups on the aromatic ring; nonetheless, the MIP showed selective recognition properties. Very likely, differently from the two carbonyl-groups on the macrocyclic ring, ZEN hydroxyl groups on the aromatic ring may form hydrogen bonds, which are responsible for interaction with MIP [19].

In this work, 4-VP, EDMA, and QUE were kept as the functional monomer, the cross-linker, and the dummy template, respectively. However, dibutyl phthalate was preferred to acetone as the porogen, and instead of bulk polymerization, which can lead to low binding capacity and poor accessibility for the template, a surface imprinting approach (consisting in the production of a MIP shell on the surface of nano and microparticles) was used [25]. This allowed preparing a magnetic material by incorporating magnetic nanoparticles into the MIP particles, thus exploiting the advantage of dispersive SPE over conventional SPE.

### 2.2. The Selectivity of the mMIP

The binding capacity of mMIP vs. that of the corresponding magnetic NIP (mNIP) was assessed previously [25]. Selectivity towards the target analyte was evaluated by observing the binding capacity of mMIP towards other ZEN-related or QUE-related compounds. These tests were carried out with a single compound each time, thus to avoid binding site saturation. As shown in Table 1, the mMIP showed to be able to bind not only ZEN, but also DAD (daidzein), genistein (GEN), β-ZEL, α-ZEL, and ZAN, whereas DON (deoxynivalenol) was not retained at all and H-T2 toxin (H-T2) only in a small amount (ca. 20%). Therefore, the mMIP could be used to monitor ZEN metabolites too. Of course, the choice of a flavonol as dummy template makes the material selective also for the two tested isoflavones DAD and GEN.

### 2.3. Optimization of Sample Clean-Up Using the mMIP

The enrichment and clean-up method based on mMIP adsorption was optimized following the literature procedure [18]. Initially, 100 mg mMIP material was washed with 5 mL acetonitrile and conditioned with 5 mL water. Then, to reproduce the extraction procedure, 3 mL acetonitrile/water 75:25 (v/v) containing 18.75 ng ZEN standard were diluted with water up to obtain a mixture water/acetonitrile 80:20 (v/v). After sample loading, a washing step was initially tested, using 2 mL of water, followed by 2 mL of acetonitrile and 2 mL of a mixture acetonitrile/methanol, 93:7 (v/v). Elution was achieved with 3 mL of methanol (three times). All the organic solutions, including loading and washing, were collected, and after solvent evaporation, the residues were reconstituted with 500 μL of methanol/water 85:15 (v/v) containing 5 mmol L^−1^ ammonium formate and 0.1% HCOOH.

An elution curve was constructed, indicating good retention of ZEN during loading (only 5% was detected in the loading phase), but also elution in the washing steps with organic solvents (75% recovery, RE, in the pooled washing 2 and 3 fractions). Only 12% of ZEN was recovered in the elution step. Given the above, the washing steps were reduced to single washing with water to avoid analyte loss, which improved the recovery by up to 76% in the optimized conditions.

The single washing step with water allowed to develop a simple and fast procedure; however, when applied to clean-up of cereal sample extracts, it could be unsuitable for removing matrix components. The consequence of unspecific interactions of the mMIP material with matrix components could be a high ME during HPLC-MS/MS analysis, thus reducing method accuracy and LOQ. Therefore, experiments on cereal samples were performed. The literature procedure [18] was further modified. A durum wheat sample fortified with 18.75 ng (i.e., 75 ng g^−1^) of ZEN was added with 2.5 mL of acetonitrile/water 80:20 (v/v) containing 0.2% HCOOH. After incubation with 100 mg of mMIP, the extract was diluted with 7.5 mL of water to obtain a final composition water/acetonitrile 80:20 (v/v). Then, the sample was treated, as described in the optimized procedure (see Experimental section). In this way, RE (extraction recovery) was only 68%. A further improvement (RE 76%) was obtained in the final condition, i.e., by diluting the extract with 22.5 mL water to obtain a final composition water/acetonitrile 92:8 (v/v), which likely resulted more favorable to mMIP-ZEN interaction. Indeed, methanol and acetonitrile were used for final elution of ZEN from mMIP; therefore, a high amount of organic solvent in this step could prevent complete absorption of the analyte by the material. The ME was negligible (97%), confirming that the water-based washing step is enough to remove most of the not retained matrix components.

The time of incubation of mMIP in the diluted extract (15 min) was chosen based on previous work [25], showing that the dynamic re-binding of the mMIP material was quick and occurred within 10 min contact.

Another parameter taken into account in method development was the composition of the reconstitution phase for the final residue, consisting of 250 µL of methanol/water, 80:20 (v/v) with 5 mmol L^−1^ ammonium formate. First, acetonitrile was chosen as the organic solvent. For 50% acetonitrile in the reconstitution mixture, a low RE was obtained, likely due to solubility problems (Table 2). By increasing the acetonitrile amount up to 80%, however, broadening of the chromatographic peak occurred.

### 2.4. Method Performance and Single-Lab Validation

#### 2.4.1. Carry-Over

Carry-over of analytes was randomly observed after injection of standard solutions at high concentration. Therefore, to prevent this phenomenon, the autosampler needle washing solution, which consisted of isopropanol/water 80:20 (v/v), was added with 0.1% (v/v) HCOOH. Furthermore, blank injections were inserted every five sample injections to verify the absence of carry-over.

#### 2.4.2. Extraction Recovery and Matrix Effect

Table 3 shows RE and ME values estimated at three different spiking levels in six replicate experiments. The lower RE observed at the highest spiking level could be ascribable to mMIP binding site saturation. Indeed, as confirmed by the cross selectivity tests, some phytochemicals could bind to the mMIP because the dummy template, employed for mMIP preparation, belonged to the flavonoid class, therefore, decreasing ZEN retention at high concentration. Nonetheless, the method is suitable for monitoring ZEN presence at the lower levels, and the bias at high concentration could be bypassed by increasing the amount of mMIP employed for clean-up.

Considering the overall process efficiency (PE) of the developed procedure (see Equations (3) and (4)), for the two lower concentration levels, it was > 95%, whereas it decreased below 80% for the highest concentration level.

#### 2.4.3. Trueness and Precision

Trueness was estimated as ZEN apparent recovery (RE_app_, see Equation (5)), by normalizing its peak area to internal standard (IS) peak area. For precision assessment, repeatability and intermediate precision were estimated by the corresponding RSD_r_ and RSD_R_, respectively. Values are reported in Table 4.

#### 2.4.4. Linearity, Limit of Detection, and Limit of Quantification

The six point-calibration curve constructed in neat standard solutions showed that the response was linear over the range 5–300 pg µL^−1^. The coefficient of determination R^2^ was 0.9948 (see Supplementary Figure S1).

The LOD and LOQ values (Table 5) were estimated according to Equations (6) and (7) reported in the Experimental section.

### 2.5. Comparison with Two Literature Procedures

The mMIP performance in sample clean-up was compared with that of a commercial OASIS HLB (Waters, Milford, MA, US) SPE column [16] and with that of a procedure without any clean-up step [26]. The two literature methods were adapted for a reliable comparison with the developed method. To this aim, the fortified samples (0.250 g durum wheat flour added with 18.75 ng ZEN, i.e., 75 ng g^−1^) were extracted with 2.5 mL acetonitrile/water 80:20 (v/v) with 0.2% HCOOH. For the procedure without clean-up, the extract was directly evaporated, and the residue reconstituted using the optimized phase (methanol/water 80:20, v/v, with 5 mmol L^−1^ ammonium formate), filtered, and injected. For the OASIS HLB clean-up, the extract was diluted with 37 mL water (final acetonitrile amount 5%) and loaded onto the preconditioned column. After washing, ZEN was eluted by 4 mL of acetonitrile, and the resulting solution treated as above (i.e., the solvent was removed, and residue reconstituted with the optimized phase). Without clean-up, RE was 98%; however, the ME signal enhancement was 208%, making the quantification unreliable, whereas with the OASIS HLB clean-up, RE was 91% and ME 125%. Results show that cereals are complex matrices, which require a clean-up step to improve method trueness. Moreover, the comparison indicated that the mMIP used in this work has a performance slightly better than a popular commercial SPE column, with the advantage of being used in a simpler dispersive SPE procedure due to its magnetic properties.

### 2.6. Cereal Sample Analysis

Finally, some cereal flour samples of different typologies (two samples for each typology) were analyzed with the developed procedure. In Table 6, for each typology, the sample with the highest detected ZEN level is reported.

## 3. Conclusions

The magnetic material mMIP, constituted by a multishell structure, was previously prepared, characterized, and the selectivity was evaluated only from ZEN standard solution. In the present work, we wanted to test the applicability of the material for the selective extraction and clean-up of ZEN from different flours, which could contain the mycoestrogen. Considering that ML for ZEN in most cereals is 75 ng g^−1^, the obtained LOQ value, 0.14 ng g^−1^, is suitable to monitor the mycotoxin contamination even at trace level in complex cereal samples.

The developed method based on the mMIP clean-up was compared with a commercial SPE copolymer and with a procedure not involving the clean-up step. The result showed the better performance of the proposed mMIP, in particular, concerning ME.

## 4. Materials and Methods

### 4.1. Chemicals and Materials

Organic solvents of analytical grade, formic acid, ammonium formate, as well as LC-MS grade methanol and ultrapure water (resistivity 18.2 MΩ cm) were obtained from Sigma-Aldrich now Merck (Darmstadt, Germany).

The authentic standards (purity ≥ 99%) of ZEN (CAS n. 17924-92-4), ZAN (CAS n. 5975-78-0), α-ZEL (CAS n. 36455-72-8), β-ZEL (CAS n. 71030− 11-0), DAD (CAS n. 486-66-8), GEN (, CAS n. 446-72-0), QUE (CAS n. 6151-25-3), HT-2 (CAS n. 26934-87-2), and DON (CAS n. 51481− 10-8) were purchased from Sigma-Aldrich (Darmstadt, Germany). Deuterated ZEN (ZEN-d6) was acquired from Wellington Laboratories (Toronto, ON, Canada) and used as IS. For each analyte, an individual stock standard solution at 200 μg mL^−1^ concentration was prepared by dissolving the suitable amount of standard in methanol. All the solutions were stored in the dark at −20 °C and brought to room temperature before use.

### 4.2. Preparation of the mMIP and Assessment of Its Binding Properties

The mMIP was prepared, as described in previous work, using QUE as a dummy template [25]. The multishell structure was constituted by a magnetic core of magnetite (Fe_3_O_4_) nanoparticles covered by a thin silica layer, surrounded by the MIP shell. The bulk polymerization was carried out using 4-VP as the functional monomer, EDMA as a cross-linker, and dibutyl phthalate as porogen.

The mMIP was extensively washed by methanol/CH_3_COOH, 90:10 (v/v) (1 mL every 100 mg material, ten times) and finally methanol (1 mL, once). Washings were repeated to obtain a residual QUE concentration in the alcoholic supernatant below 0.1 ng mg^−1^ material.

Re-binding evaluation experiments showed that the mMIP had 55-fold more affinity for ZEN than the corresponding mNIP [25].

### 4.3. Sample Preparation

Different cereal flours were acquired in local markets of Rome (Italy), namely maize, durum wheat, hulled wheat, whole wheat, tapioca and maize mix, rice, and buckwheat, and extracted according to La Barbera et al. [27] with some modifications. Briefly, 250 mg flour were weighed in a 15 mL-polypropylene centrifuge tube and, for experiments requiring artificial contamination (such as RE and ME assessment), was soaked in 250 µL acetone spiked with the suitable amount of ZEN standard solution, then left to dry in the fume hood for 15 min to favor analyte diffusion through the solid sample. After this step, 2.5 mL of an extraction mixture, constituted by acetonitrile/water, 80:20 (v/v) with 0.2% HCOOH, was added and the sample vortexed for 5 min and sonicated for 10 min. After that, the extract was recovered by centrifugation at 9000× *g* at 4 °C to allow lipids and flour precipitation.

The extract was put in a 50 mL-polypropylene centrifuge tube and added with 22.5 mL of ultrapure water (final composition water/acetonitrile 98:2, v/v) and 100 mg of mMIP. The resulting solution was first vortexed for 15 min at a low rate to allow the interaction between the material and the analyte, then centrifuged at 9000× *g* at 4 °C for 15 min. By the aid of an external magnet (a permanent magnetic disk Nd-Fe-B, 25 mm × 5 mm, Supermagnete, Gottmadingen, Germany), the solution was decanted, and the supernatant was removed. After that, the mMIP, with the analyte adsorbed on it, was moved to a 2 mL polypropylene microcentrifuge tube and washed with 2 mL of water, vortex mixed for 5 min and centrifuged at 9000× *g* for 2 min. After removal of the supernatant, ZEN was eluted by 1 mL of methanol (three times) and finally 1 mL of acetonitrile (two times). Each time, the solution was mechanically mixed for 3 min, centrifuged 2 min at 9000× *g*, and finally, the mMIP was allowed to settle down by magnetic decantation.

The pooled eluate was evaporated by a gentle nitrogen stream in a water bath at 37 °C; the residue was reconstituted with 250 µL of methanol/water, 80:20 (v/v) containing 5 mmol L^−1^ ammonium formate. After a centrifugation step (18,000× *g* for 5 min at 20 °C by a MicroCL 21R centrifuge, Thermo Scientific, Waltham, MA, USA), the supernatant was passed through a 13 mm GHP membrane syringe filter (0.2 μm, Pall Corp., Port Washington, NY, USA) and transferred into an autosampler polypropylene vial. Ten microliter aliquots were injected in the HPLC-MS system.

### 4.4. UHPLC-MS/MS Analysis

The LC-MS/MS instrumentation consisted in a UHPLC system Ultimate 3000 binary pump (Thermo Fisher Scientific, Bremen, Germany) coupled to a triple quadrupole mass spectrometer (TSQ Vantage EMR, Thermo Fisher Scientific, Bremen, Germany) via a heated electrospray (ESI) source. The software XcaliburTM v.2.2 (Thermo Fisher Scientific, Bremen, Germany) was used for managing LC-MS data acquisition and processing.

Analytes were separated onto a CORTECS UPLC C18 column (2.1 mm i.d. × 100 mm, 1.6 µm particle size, 90 Å pore size) equipped with a CORTECS UPLC C18 + guard column (2.1 mm i.d. × 5 mm, 1.6 µm, 90 Å pore size), maintained at 40 °C and operating at a flow-rate of 0.3 mL min^−1^. The chromatographic mobile phase was water (A) and methanol (B), both containing 5 mmol L^−1^ ammonium formate and 0.1% HCOOH. The following gradient was used for analytes elution: after a 30 s isocratic step at 15%, B was linearly increased to 35% in 1 min, then to 68% in 3.5 min, to 75% in 3 min, and finally to 98% in 1 min. The column was washed at 98% B for 3 min and then equilibrated at 15% B for 6 min.

Mass spectra were acquired using polarity switching in multiple reaction monitoring (MRM) mode. The tune parameters were set as follows: spray voltage, +3.2/−2.8 kV; vaporizer temperature, 280 °C; capillary temperature, 220 °C; sheath gas pressure, 50 (arbitrary units, a.u.); sweep ion gas pressure (+) 0/(−) 1 (a.u.); auxiliary gas pressure, 25 (a.u.). For each compound, at least two MRM transitions were monitored (see Table S1 of Supplementary Material). Monthly, the calibration solutions provided by Thermo Fisher Scientific (*m/z* 69-2800) were injected in infusion mode for mass calibration and resolution adjustments of the resolving lens and quadrupole.

### 4.5. The Selectivity of MIP Towards ZEN

The selectivity of mMIP towards ZEN was assessed by incubating 100 mg of the material with single standard solutions of ZEN, α-ZEL, β-ZEL, ZAN, DAD, GEN, DON, and H-T2, at 10 ng µL^−1^, in water/acetonitrile 90:10 (v/v). Analytes and the mMIP were left 30 min under mild mechanical mixing condition. Then, the supernatant was collected by magnetic decantation and analyzed. The presence of analytes in the supernatant indicated a scarce affinity for the material, while the analytes, which were not detected, demonstrated a high affinity for the mMIP.

### 4.6. Method Validation 

Single-lab method validation was carried out following the main validation guidelines, and following the suggestions by Kruve et al. [28,29]. The considered performance parameters were the linear range, LOD and LOQ, trueness, intra-day and inter-day precision, and RE and ME.

#### 4.6.1. Extraction Recovery and Matrix Effect

RE and ME were estimated, as reported in other papers [27,30]. For this purpose, three different solutions were used, namely, a blank matrix spiked with the standard analyte before (set 1) and after (set 2) the mMIP clean-up step, and neat standard solutions (set 3). As a blank matrix, a durum wheat sample previously checked to be analyte-free, i.e., naturally contaminated at level < LOD, was used. Absolute peak areas (i.e., without normalization to IS) were measured.

RE and ME were assessed by the ratio between the peak area of two different sample sets, according to the equations:

RE% = (Area_set1_/Area_set2_) × 100 (1)

ME% = (Area_set2_/Area_set3_) × 100. (2)

The overall PE can be obtained by applying one of the two following equations:

PE% = (Area_set1_/Area_set3_) × 100 (3)

PE% = RE × ME.(4)

The experiments were performed using three different spiking levels for ZEN. For matrix solutions, considering that the ML fixed for most cereals different from maize is 75 ng g^−1^, spiking levels were 0.5 × ML (37.5 ng g^−1^), 1.0 × ML (75 ng g^−1^), and 4.0 × ML (300 ng g^−1^). For standard solutions, the corresponding levels were 37.5, 75, and 300 pg µL^−1^. Six replicates for each concentration level were performed.

#### 4.6.2. Linear Range and Calibration Curve

A six-point standard calibration graph was constructed for ZEN. Standard solutions were prepared by diluting the standard working solution with suitable volumes of methanol/water 80:20 (v/v) containing 5 mmol L^−1^ ammonium formate. The six points were 5, 30, 50, 75, 150, and 300 pg µL^−1^. Each solution was prepared in duplicate and injected twice, starting from the lowest up to the highest concentration level; finally, the results were averaged to produce a single calibration graph. 

The combined ion current profile for the two selected transitions was extracted from the LC-MRM dataset; the resulting traces were smoothed (Gaussian type, 7 points) by applying the automatic processing smoothing of Xcalibur^TM^ 2.2 software. ZEN peak area versus its concentration was plotted, and the unweighted regression line for calibration graph was calculated using Xcalibur^TM^ QuanBrowser (Thermo Fisher Scientific, Bremen, Germany).

#### 4.6.3. Trueness and Precision

For trueness experiments, ZEN peak area was normalized to the IS peak area. The RE_app_ was calculated by comparing ZEN to ZEN-d6 peak area ratios in analyte-free flour samples spiked before and after the extraction procedure, following the equation:

RE_app_% = [(Area analyte_set 1_/Area IS_set 1_)/(Area analyte_set 2_/Area IS_set 2_)] × 100 (5)

Fortification levels were the same used for RE experiments. IS was added at the same amount, i.e., 150 pg µL^−1^. Also, in this case, six replicates for each fortification level were considered and averaged.

Within the laboratory, the precision was estimated as intra-day (repeatability) and inter-day (intermediate precision) precision. For this purpose, the RSD (relative standard deviation) of the RE_app_ of six spiked samples at 0.5 × ML concentration analyzed in the same day (RSDr) and in six consecutive days (RSD_R_) were considered.

#### 4.6.4. LOD and LOQ

For the estimation of LOD and LOQ values, durum wheat flour samples, whose ZEN contamination was < LOQ, were used. The values were obtained by averaging the results of six replicates and considering the most intense MRM transition (quantifier transition: m/z 317.1 → m/z 131.1) to estimate LOQ and the less intense MRM transition (qualifier transition: m/z 317.1 → m/z 175.1) to estimate LOD.

A signal-to-noise ratio (S/N) = 3 for the second most intense transition and S/N = 10 for the most intense transitions were extrapolated from the MRM dataset and used in the following equations:

LOD = 3 × S/N(6)

LOQ = 10 × S/N(7)

## Figures and Tables

**Table 1:** The binding capacity of the mMIP (magnetic molecularly imprinted polymer) towards ZEN (zearalenone) and other compounds, with relative chemical structures.

Compound	Structure	Not Retained Amount (RSD, %) ^1^	Compound	Structure	Not Retained Amount (RSD, %) ^1^
DON	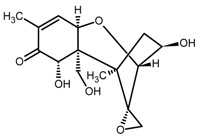	112% (14)	β-ZEL	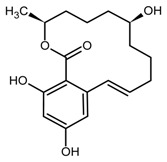	3% (2)
DAD	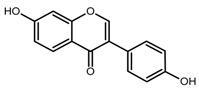	5% (3)	α-ZEL	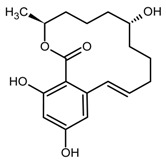	<LOD
GEN	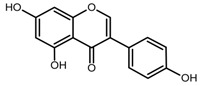	<LOD	ZEN	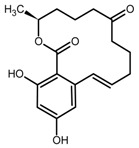	4% (2)
H-T2	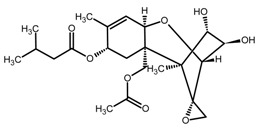	81% (9)	ZAN	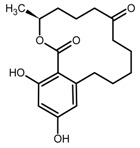	<LOD

^1^ Compound percent remained unbound in the supernatant solution after incubation with mMIP, as described in Section 4.5, with the related relative standard deviation (RSD, n = 3).

**Table 2:** Extraction recovery (RE) and matrix effect (ME) for the compositions tested for reconstitution of the residue before liquid chromatography-mass spectrometry analysis.

Compound	Acetonitrile/Water 50:50 (v/v) RE; ME (%) ^1^	Acetonitrile/Water 80:20 (v/v) RE; ME (%) ^1^	Methanol/Water 80:20 (v/v) RE; ME (%) ^1^
ZEN	80; 86	92; 85	95; 99

^1^ ZEN fortification level was 75 ng g^−1^; all the solutions contained 5 mmol L^−1^ ammonium formate.

**Table 3:** Extraction recovery (RE) and matrix effect (ME).

Compound	RE% ^1^ (RSD) 0.5 × ML	ME% ^1^ (RSD) 0.5 × ML	RE% ^1^ (RSD) 1.0 × ML	ME% ^1^ (RSD) 1.0 × ML	RE% ^1^ (RSD) 4.0 × ML	ME% ^1^ (RSD) 4.0 × ML
ZEN	98 (7)	99 (3)	94 (3)	102 (6)	76 (9)	103 (4)

^1^ RE and ME were estimated by applying the Equations (1) and (2) described in the Experimental section. Relative standard deviations (RSD, n = 6) are reported.

**Table 4:** Trueness and precision.

Compound	RE_app,_ % (RSD_r_; RSD_R_)		
**Spiking level**	**0.5 × ML**	**1.0 × ML**	**4.0 × ML**
ZEN	98 (10; 7)	97 (8; 11)	81 (9; 13)

**Table 5:** Limit of detection (LOD) and quantification (LOQ).

Compound	LOD (ng g^−1^)	LOQ (ng g^−1^)
ZEN	0.044	0.14

**Table 6:** Cereal flour sample analysis.

	Flour Sample Contamination (ng g^−1^)						
Compound	Tapioca and Maize	Maize	Hulled Wheat	Whole Wheat	Rice	Buck Wheat	Durum Wheat
ZEN	7.5	8.5	8.9	9.7	15.0	<LOQ	<LOQ

## Supplementary Materials

The following are available online at https://www.mdpi.com/2072-6651/11/9/493/s1, Figure S1: Six point-calibration curve constructed in neat standard, Table S1: Acquisition parameters for tandem mass spectrometry analysis.
