# Supplementary material for: A Novel Magnetic Molecular Imprinted Polymer for Selective Extraction of Zearalenone from Cereal Flours before Liquid Chromatography-Tandem Mass Spectrometry Determination
Source: Toxins (Basel). 2019 Aug 27;11(9):493. doi: 10.3390/toxins11090493 (PMC6784151; doi:10.3390/toxins11090493)
Supplement: Supplementary file 1 [file toxins-11-00493-s001.pdf]

Supplementary Materials: A Novel Magnetic Molecular Imprinted Polymer for Selective Extraction of Zearalenone from Cereal Flours before Liquid Chromatography-Tandem Mass Spectrometry Determination

Chiara Cavaliere, Michela Antonelli, Andrea Cerrato, Giorgia La Barbera, Aldo Laganà, Michele Laus, Susy Piovesana and Anna Laura Capriotti

Table 1. Acquisition parameters for tandem mass spectrometry analysis.

Compound	Acronym	Retention Time (*min*)	Polarity	Precursor Ion (*m/z*)	Product Ions, *m/z* (Collision Energy, *V*)	S-LENS (*V*)
Deoxynivalenol	DON	4.2	-	341.0	265.0 (11)<br>295.0 (11)	65
Quercetin	QUE	5.9	+	303.0	153.1 (32)<br>137.1 (32)<br>165.0 (26)	150
Daidzein	DAD	8.5	+	255.0	137.1 (27)<br>199.1 (24)	170
Genistein	GEN	9.2	+	271.0	91.3 (36)<br>153.1 (27)	120
H-T2-Toxin	H-T2	9.3	+	442.2	215.1 (12)<br>263.1 (12)	95
Zearalenone	ZEN	10.2	-	317.1	131.1 (33) *Q*[1]<br>160.0 (34)<br>175.1 (25) *q*[1]	135
ß-Zearalenol	β-ZEL	10.4	-	319.2	160.0 (28)<br>275.1 (21)	135
α- Zearalenol	α-ZEL	10.7	-	319.2	160.0 (32)<br>275.1 (21)	135
Zearalanone	ZAN	10.9	-	319.2	61.0 (30)<br>205.0 (20)	135
Deuterated Zearalenone	ZEN-d6 (IS)	10.2	-	323.0	131.1 (33)<br>175.1 (25)	140

[1]*Q*: quantifier MRM transition; *q*: qualifier MRM transition.

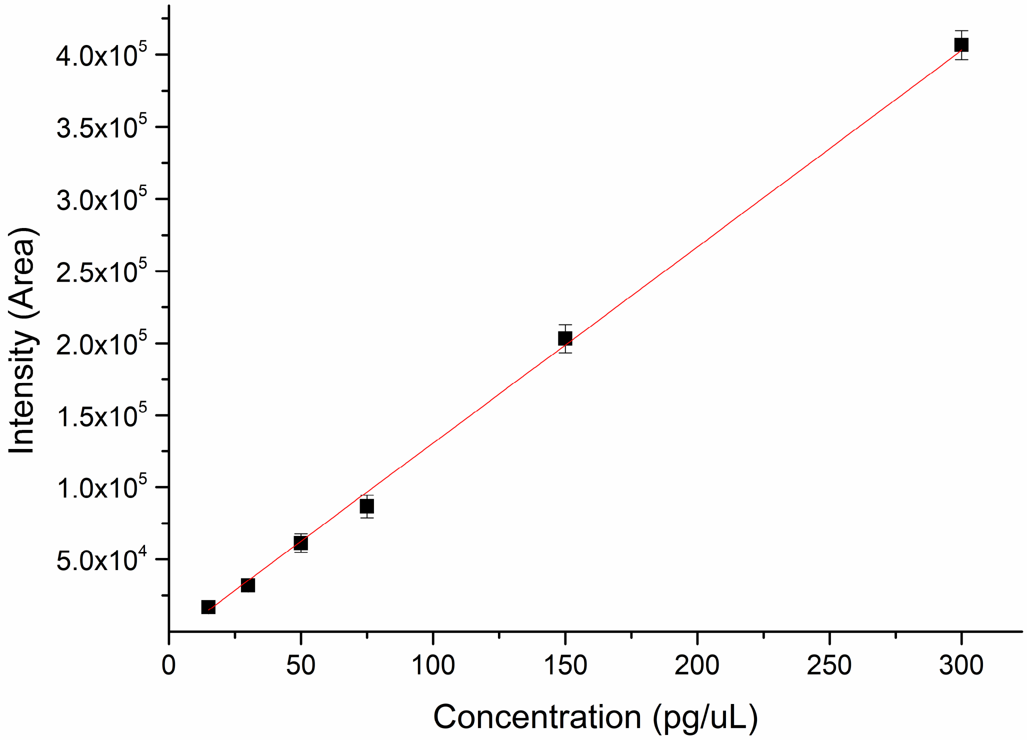

Figure 1. Six point-calibration curve constructed in neat standard.
The resulting equation is: y = 1390.3 x -8985.6; R^2 = 0.9948.
